# Two Simple Methods for Optimizing the Production of "Difficult-to-Express" GnRH-DFF40 Chimeric Protein

**DOI:** 10.15171/apb.2019.050

**Published:** 2019-08-01

**Authors:** Mahdi Barazesh, Zohreh Mostafavipour, Soudabeh Kavousipour, Shiva Mohammadi, Pooneh Mokarram

**Affiliations:** ^1^Department of Biotechnology, School of Advanced Medical Science and Technologies, Shiraz University of Medical Sciences, Shiraz, IR Iran.; ^2^Department of Biochemistry, School of Medicine, Shiraz University of Medical Sciences, Shiraz, IR Iran.; ^3^Recombinant Proteins Lab, School of Medicine, Shiraz University of Medical Sciences, Shiraz, IR Iran.

**Keywords:** Humanized recombinant immunotoxin, GnRH-DFF40 chimeric protein, Autoinduction method (AIM), High cell density IPTG induction (HCDI), Targeted therapy

## Abstract

***Purpose:*** GnRH-DFF40 (gonadotropin releasing hormone - DNA fragmentation factor 40) is a humanized recombinant immunotoxin and serves as a prospective candidate for targeted therapy of gonadotropin releasing hormone receptor (GnRHR) overexpressing malignancies. However, its production in Escherichia coli in a soluble and functional form still remains a challenge. Here we introduce two successful and reproducible conditions for production and purification of “difficult-to-express” GnRH-DFF40 protein.

***Methods:*** A synthetic codon optimized GnRH-DFF40 fusion gene was cloned in pET28a plasmid. Two methods including high cell density IPTG induction (HCDI) and autoinduction method (AIM) with a focus on obtaining high cell density have been investigated to enhance the protein production in (E. coli). Moreover, to obtain higher protein production several factors in the AIM method including carbon sources, incubation time and temperature, plasmid stability and double colony selection, were optimized.

***Results:*** Remarkable amounts of soluble GnRH-DFF40 protein were achieved by both methods. Cell density and protein yields in AIM was about 1.5 fold higher than that what obtained using HCDI. Initial screening showed that 25ºC is better to achieve higher protein production in both methods. pH alterations in AIM were maintained in a more constant level at 25ºC and 37ºC temperatures without any detrimental effects on cell growth during protein production phase up to 21 hours after incubation. Plasmid stability during growth and expression induction phase was maintained at a high level of 98% and 96% for AIM and HCDI methods, respectively. After parameter optimization and double colony selection in AIM, a very high yield of recombinant protein was achieved (528.3 mg/L).

***Conclusion:*** With the optimization of these high cell density expression methods, reproducible manifold enhancement of soluble protein yields can be achieved for “difficult-to-express” GnRH-DFF40 compared to conventional expression methods.

## Introduction

Cancer cells can persist against chemotherapeutic agents by several mechanisms including resistance to apoptotic signals, up-regulation of anti-apoptotic machinery and mutation or down-regulation of apoptotic components such as DFF40 (DNA fragmentation factor 40) nuclease.^1^ Also, the lack of selectivity in chemotherapy approaches has led to the development of targeted therapy methods that efficiently deliver a humanized apoptotic agent to the cancer cells.^2,3^ GnRH-DFF40 (gonadotropin releasing hormone - DNA fragmentation factor 40) is a humanized recombinant immunotoxin which is composed of a cell-targeting delivery moiety (GnRH or gonadotropin releasing hormone) and a killing agent, DFF40. This immunotoxin is capable to remove selectively cancerous cells overexpressing gonadotropin releasing hormone receptor (GnRHR) without affecting the healthy adjacent cells.^4^ GnRH, a decapeptide hormone, play a role in targeted transmembrane delivery moietyw of different molecule cargos such as polypeptides, proteins and nanoparticles and can efficiently deliver its apoptotic fusion partner (DFF40) into tumor cells via interaction with its receptor which is overexpressed in the surfaces of solid tumors and hormone-responsive cancer cells with no or little systemic side effects, toxicity and immunogenicity compared with immunotoxins with non-human origins, such as bacterial or plant toxins.^5,6^ DFF40 or caspase activated DNAse (CAD), is a human cysteine-rich protein with double-strand-specific endonuclease activity that can solely and directly trigger DNA fragmentation in the final stage of apoptosis.^7,8^ Since GnRH receptor is poorly expressed on normal cells, and on the other hand DFF40 is mutated or deleted in many types of cancers it is an attractive approach for the targeted reintroduction of DFF40 to these malignancies. Recent *in vitro* and *in vivo* studies in colon adenocarcinoma proved this capability.^9^

*Escherichia coli* is one of the widely used expression systems. Due to its low cost, simplicity, achieving high cell densities in a short time made it as an affordable choice and robust system for the production of recombinant proteins both in laboratory and High-throughput scale.^10^ However, GnRH-DFF40 is difficult to produce in *E. coli.* The existence of 11 cysteine residues and consequently disulfide bond formation within DFF40 and biologically inactive inclusion body formation with low efficiency in its refolding and purification, are some challenging approaches.^6,11^ Other factors related to this problem include its cytotoxicity to the host cells, rare codon usage, the stability and translational efficiency of cloned gene in the plasmid, remarkable pH reduction in the culture medium, the proper folding facility of the protein, and its degradation by host proteases.^6^ One important strategy is the optimization of cell growth conditions and media components to overcome these limiting factors. In this study, autoinduction (AIM) and high cell density IPTG induction (HCDI) methods as simple strategies were used to achieve a high yield of synthetic codon optimized and soluble form of GnRH-DFF40 heterologous protein in *E. coli*. These techniques with a focus on obtaining high cell density and simultaneously enhancing buffering capacity and plasmid stability, improve soluble protein production yields. Thus, we aimed to discover the best condition for successful and reproducible soluble GnRH-DFF40 production and purification as a novel humanized therapeutic immunotoxin by these two affordable approaches.

Determination of optimum condition for protein production by different glucose and lactose concentrations using the highest expression colony obtained from the double selection at 25°C temperature and after 21 h incubation time. Data are represented as mean protein concentration ± SEM. ± indicates the standard error mean of three independent experiments, performed for reproducible production of the chimeric protein using autoinduction method.

## Materials and Methods

### Bacterial strains, plasmids and growth conditions

Bacterial growth materials, antibiotics, Isopropyl β-D-1-thiogalactopyranoside (IPTG), Histidine (His) tag monoclonal antibody produced in mouse, routine laboratory chemicals and disposable labware were from Sigma-Aldrich (St. Louis, MO) and Thermo Fisher (Pittsburgh PA). pET-28a, pET22b, and pGEX 4T1 plasmids were purchased from Novagen, USA. Chemically competent BL21 (DE3), BL21 (DE3, pLysS), and Rosetta (DE3) *E. coli* strains were transformed using standard protocols applied as hosts for recombinant protein expression (Novagen, USA). Host strain, *E. coli* DH5a, was used for sub-cloning and plasmid propagation (Novagen, USA). Protein concentrations were determined by the BCA (Bicinchoninic acid) protein assay kit according to the manufacturer’s instructions (Sigma-Aldrich, USA). General molecular cloning methods were done according to Sambrook et al.^12^

### Plasmid design encoding native and synthetic GnRH- DFF40

For the construction of chimeric GnRH-DFF40, the DFF40 coding sequence was amplified with specific primers using pIRES2- EGFP-DFF40 vector (Biomatik, Ontario, Canada) as a template. Then DFF40 fragment was cloned into kanamycin-resistant pET28a plasmid containing N-terminal His tag. A set of specific primer was used in order to fuse GnRH to 5ʹ end of DFF40 by overlap PCR and pET28a-DFF40 as template (Table 1). The chimeric fragment (GnRH-DFF40) was cloned into pET28a (+) vector between NdeI and SalI restriction site and then sub-cloned into ampicillin-resistant pET22b and pGEX 4T1 expression vectors (containing N-terminal His tag and GST tag respectively) with the same restriction sites. To confirm the successful cloning and proper direction of the inserted gene, double digestion with NdeI and SalI enzymes and PCR analysis using T7 promoter-specific primers were done, respectively. Sequence identity of the cloned fragment was also confirmed by DNA sequencing. Codon optimization was done according to the codon bias of *E. coli* genes using gene script database (https://www.genscript.com/codon-opt.html) and finally, codon-optimized gene was synthesized by Biomatik Company (Ontario, Canada).

**Table 1:** List of forward and reverse primers

**Primer**	**Sequence**	**Annealing temperature (°C)**	**Product size (bp)**
DFF40 F	5^´^TTTCATATGCTCCAGAAGCCCAAGAG 3	60	1020
DFF40 R	5^´^ TTTGTCGACCTGGCGTTTCCGCACAGGC 3´	60	1020
GnRHDFF40 F	5^´^TTTCATATGGAGCACTGGTCCTATGGACTGCGCCCTGGAATGCTCCAGAAGCCCAAGAG 3´	62	1065
DFF40 R	5^´^ TTTGTCGACCTGGCGTTTCCGCACAGGC 3´	62	1065

Two pairs of designed primers used for PCR amplification and cloning of DFF40 (DNA fragmentation factor 40) and GnRH-DFF40 (gonadotropin releasing hormone - DNA fragmentation factor 40) respectively. The underlined sequences show restriction digestion sites. F and R are instead of Forward and Reverse primers respectively.

### Genetic stability of recombinant plasmid and double colony selection

To investigate the stable inheritance of recombinant plasmid-carrying cells to the progenies and double selection of high expression-colonies, samples were taken from all culture conditions during the post-induction of expression in *E. coli*. In Brief, samples were spread onto the non- selective Luria-Bertani (LB) agar plates and incubated at 37°C overnight. Next day, at least 50 individual colonies picked randomly, were replica-plated on selective LB agar plates containing appropriate antibiotics: 50 μg/mL kanamycin or 100 μg/mL ampicillin and nonselective control plates simultaneously by using sterile toothpicks. Plasmid stability index (%) was calculated using the formula [(50-N)/50] x100, where N is the number of colonies formed on the kanamycin or ampicillin LB agar plates. Also, to evaluate the protein expression, several colonies provided by double colony selection in AIM and HCDI methods were randomly picked up. Afterward, highly expression-colony was used for further optimization in the AIM method.

### Recombinant protein expression with different routes

**Figure 1:**
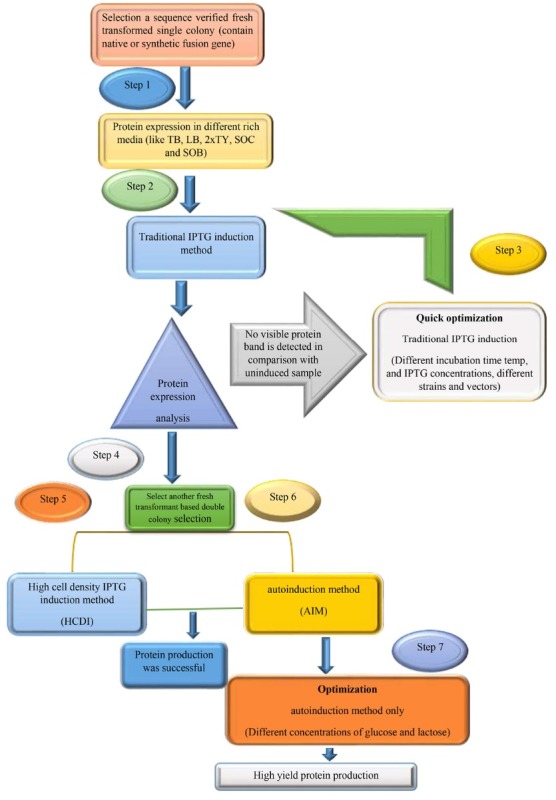
A schematic diagram of the different strategies used in the present work for successful production and achieving a very high yield of GnRH-DFF40 recombinant protein.

The target fusion gene was expressed in two formats (native and synthetic codon-optimized gene) after transformation of the constructed recombinant plasmids containing GnRH-DFF40 into chemically competent *E. coli* strains BL21 (DE3), BL21 (DE3, pLysS) and Rosetta (DE3), as summarized in Figure 1. Finally to achieve very-high-yield of recombinant protein expression, these two efficient, simple and affordable methods using pET28a were used and compared.

### Media formulations and expression conditions in AIM method

The non-inducing and AIM stock solutions were prepared according to the method originally defined by Studier with slight modifications.^13^ For AIM expression, a sequence-verified single bacterial colony obtained from double selection was grown in 5 mL of basic medium containing 50 μg/mL kanamycin and incubated overnight at 37°C. The next day, 1 mL of this media was diluted 1:100 in a 500 mL flask containing 50 μg/mL kanamycin and incubated overnight. Expression was done with two temperatures (37°C and 25°C). The pH of the culture medium and cell density (OD600) were monitored during the incubation period every 3 hours while shaking at 250 rpm to determine the best time for achieving high cell density before detrimental effects of pH reduction. Two 500 mL flasks with the same conditions without lactose were incubated as uninduced controls at both temperatures.

### Media formulations and expression conditions in HCDI method

Similar to the AIM method, a BL21 (DE3) single colony from a freshly transformed plate obtained from double selection as the starting culture was incubated overnight in 5 mL LB medium containing 50 µg/mL kanamycin in a 50 mL flask and incubated overnight at 250 rpm, 37°C. The next day, 1 mL of the overnight culture was transferred in a 500 mL flask containing 50 mL 2x TY medium. With the OD600 of about 1-1.5, the bacterial cells were centrifuged at 5000 g for 7 minutes at room temperature. The cell pellets were gently re-suspended in 100 mL of modified minimal medium in 500 mL flask containing 5 mL of 20x phosphate buffer, 0.1% NH4Cl, 10mM NaCl, 5mM MgSO4, 0.2mM CaCl2, 0.25x trace metals solution, 0.25x vitamins, 1% glucose, 1x amino acid solutions. The cells were allowed to adopt the medium exchange for another 1.0-1.5 hours. Then, protein expression was induced by the addition of 1mM of IPTG and incubated overnight with shaking. To identify the optimum time to obtain high cell density before detrimental effects of pH reduction, the pH of the culture medium and cell density (OD600) were monitored during the incubation period every 3 hours at both 37°C and 25°C temperatures.

### Optimization of AIM method

To optimize the protein yield in AIM method, variables that often had the main effect on the amount of soluble recombinant protein in the AIM method were optimized. The influence of different glucose (0.05% to 0.2%) and lactose (0.2% to 0.7%) concentrations as two major carbon sources on cell density and protein yield were investigated. Moreover, bacterial glycerol stock prepared from double colony selection with the highest expression capability was used for starting culture overnight. In all optimization steps, the cell density (OD600) and the purified protein concentration were measured by spectrophotometer and BCA protein assay kit respectively at the end of the incubation period (21 hours at 25°C). To confirm the reproducibility of recombinant fusion protein production, all experiments were done as triplicate and in three independent tests.

### Recombinant protein expression analysis and purification

Harvested cells taken from different conditions were re-suspended in lysis buffer (50mM Tris, 200 µM phenylmethylsulfonyl fluoride (PMSF), 3mM Dithiothreitol (DTT), 2mM Magnesium chloride and 300 µg/mL lysozyme, pH 7.5). The cells were lysed and homogenized by sonication (Soni-prep 150, MSE (UK) Ltd,) at 50% amplitude for 10 cycles (10 seconds pulse on, 10 seconds pulse off) at 4°C and centrifuged 45 minutes at 20 000 g for supernatant collection. Protein purification was done using Ni-NTA affinity column (Qiagen, USA). The identity of the DFF40-GnRH fusion protein in the crude extract, flow-through, wash and elution samples were analyzed on 12% SDS-PAGE and confirmed by western blot. Briefly, after proteins separation on 12% SDS-PAGE, the gel was transferred to nitrocellulose membrane. Then, the membrane was blocked in 3% (w/v) skim milk for 1 hour at room temperature. The membrane was incubated for another 2 hours with anti-poly His monoclonal antibody. The bands were detected using color development reagent, DAB (3, 3’-diaminobenzidine) and H2O2 as substrate.

## Results and Discussion

### Plasmid constructs design and validation

**Figure 2:**
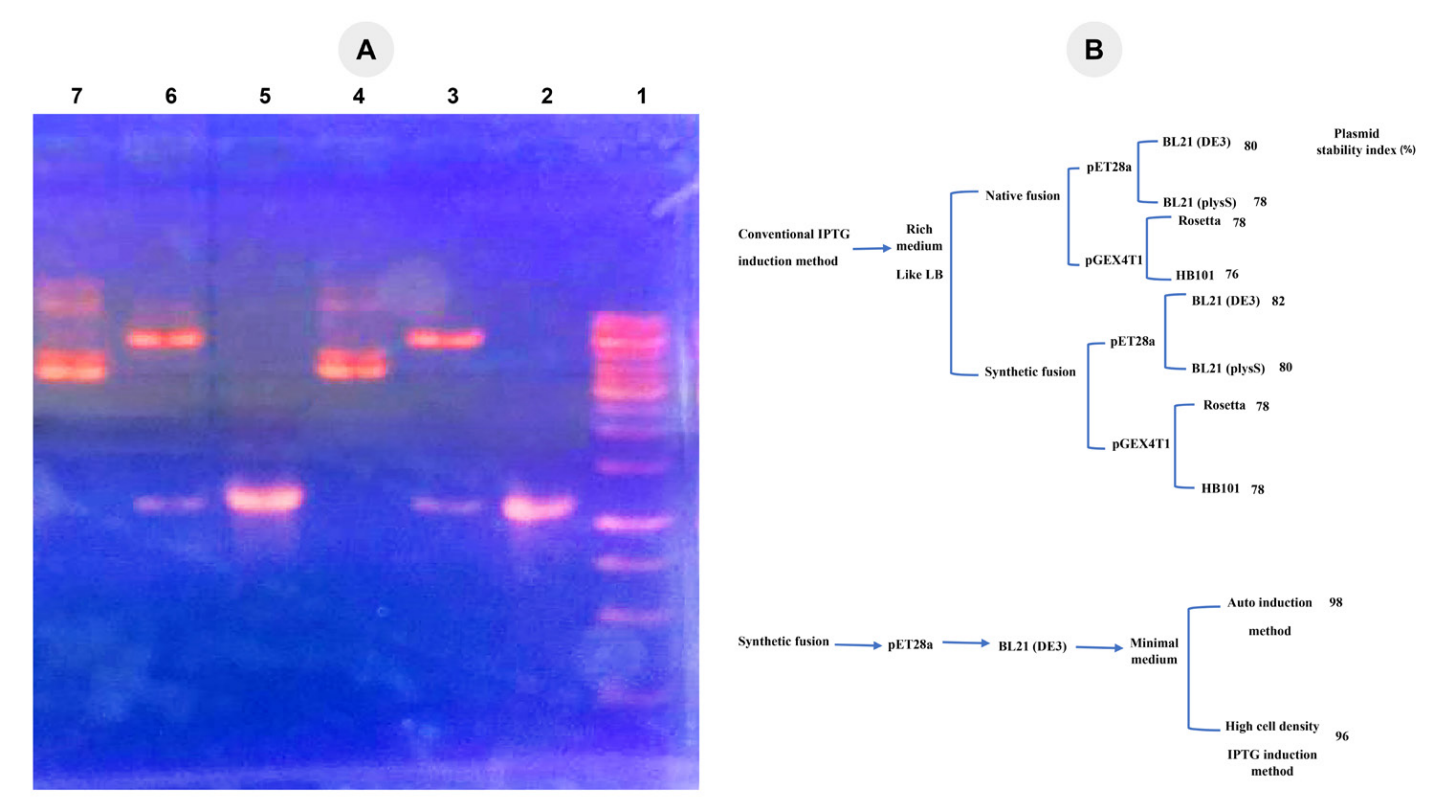
(A) PCR and double digestion analysis in order to verification of DFF40 (DNA fragmentation factor 40) and GnRH-DFF40 (gonadotropin releasing hormone - DNA fragmentation factor 40) cloning. Lane 1: molecular size marker, lanes 2 and 3: PCR amplification and double digestion for DFF40 fragment respectively, lane 4: intact vector containing cloned DFF40 fragment, lanes 5 and 6: PCR amplification and double digestion for GnRH-DFF40 fragment respectively, lane 7: intact vector containing cloned GnRH-DFF40 fragment. (B) Calculation of plasmid stability index based on the related formula for differential expression methods carried out in the present work.

Results of double digestion by the restriction enzymes (NdeI/SalI) and the final PCR product for both stages of cloning in pET28a plasmid were shown in Figure 2A. The results demonstrated that the native fusion fragment containing GnRH-DFF40 was successfully cloned in a proper direction under the control of lac promoter in pET28a expression plasmid. Sequence alignment of the cloned GnRH-DFF40 fusion displayed 100% similarity with the sequence of *Homo sapiens* GnRH and DFF40 published in GenBank.

### Codon optimization and gene synthesis

To enhance the expression of this fusion in *E. coli*, codon optimization was done according to the codon bias of *E. coli* genes using gene script database. The optimized coding sequence of GnRH-DFF40 (1065 bp) with codon adaptation index (CAI) of 0.96 was synthesized. During the codon optimization, high GC-content sequences, RNA instability structures, and other negative cis-acting elements were discarded. After optimization process, the optimized sequence and the wild-type had 70.3% similarity (In total 356 nucleotides were replaced). The low identity of native gene structure before codon optimization and subsequently low CAI^14^ led to a failure in protein production by different protocols examined in this study (Figure 1). Moreover, native fusion form had higher GC content that was adjusted in the synthetic form. The G + C percentage reduced from 56.4% to 46%. Sinclair and Choy suggested that rare codon increases mRNA instability and hampered an efficient translation, while G+C rich regions could decrease translational rate or even led to expression failure.^15^

### Different strategies for expression of the recombinant fusion protein

As shown in Figure 1, the GnRH-DFF40 expression using different bacterial hosts, various vectors, the different type of media, different IPTG concentrations (0.5, 1 and 1.5 mM ), various temperatures, incubation time and finally using the native or synthetic forms of GnRH-DFF40 gene led to no significant different in protein bands compared with un-induced samples. These results demonstrated that sometimes, the successful production of eukaryotic proteins in *E. coli* host in a biologically active state is completely challenging.^16^ The presence of disulfide bonds in DFF40 molecule and its prone to aggregation identity or formation of inclusion body is a key problem.^6,17^ Besides, reductive condition of the bacterial cytoplasm and the DNase nature of DFF40 that cause cytotoxicity to bacterial host cells are other challenges.^18^ One approach to overcome these limitations is selection of an optimal medium that helps to obtain a high cell density of bacterial population and retains plasmid stability at a high rate.^19^

**Figure 3:**
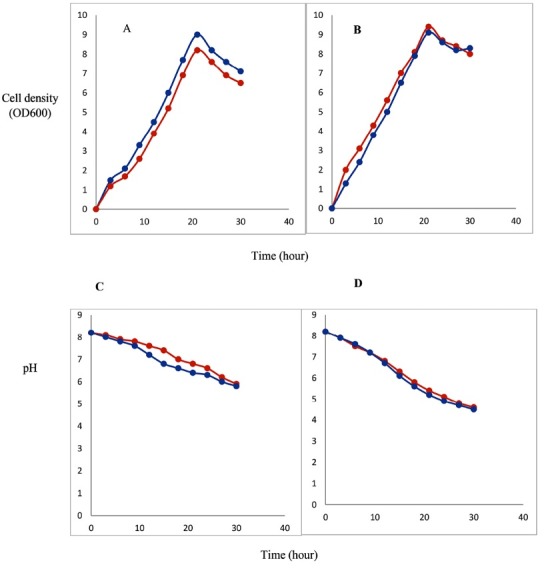
Cell density (OD 600) and pH alterations during protein expression in AIM and HCDI method. (**A**) OD (600) monitoring during the incubation period at 25°C in AIM (red line) and HCDI method (blue line). (**B**) OD (600) monitoring during the incubation period at 37°C in AIM (red line) and HCDI method (blue line). (**C**) pH monitoring during the incubation period at 25°C in AIM (red line) and HCDI method (blue line). (**D**) pH monitoring during the incubation period (30 h) at 37°C in AIM (red line) and HCDI method (blue line).

Therefore, in this study, we focused on two high-cell-density bacterial expression methods including an autoinduction (AIM) and a high-cell-density IPTG-induction (HCDI) to improve the production of “difficult-to-express” GnRH-DFF40 protein. Both methods often result in very higher amounts of soluble fusion protein compared with conventional IPTG induction methods.^20,21^ Our results indicated that both high-cell-density bacterial expression methods were successful in improvement of the heterologous protein production. However, the amount of GnRH-DFF40 protein, as well as cell density in AIM was far higher than that obtained using HCDI at both temperatures (Table 2 and Figure 3A and B). Our results confirmed direct relation between cell density and protein yields as reported by Koehn et al.^22^ Other studies showed that upon glucose depletion in AIM media, lactose consumption results in induction a high level of target protein expression at time that bacterial population has reached maximum of their cellular mass.^23,24^ On the other hand, in the lack of glucose, glycerol can reinforce growth without affecting the use of other carbon sources and pH alteration.^25^

**Table 2:** Determination of GnRH-DFF40 protein yields and cell density after 21h incubation at 37°C and 25°C temperatures in AIM and HCDI methods

**Method**	**37°C**		**25°C**	
	**Cell density (OD600)**	**Protein concentration (mg/L)**	**Cell density (OD600)**	**Protein concentration (mg/L)**
AIM	9.2	132.28	9	160.68
HCDI	9	98.6	8.1	120.38

Abbreviations: GnRH-DFF40, gonadotropin releasing hormone - DNA fragmentation factor 40 fusion protein; AIM, autoinduction method; HCDI, high cell density IPTG induction method; OD600 (optical density at 600 nm wave length).

**Figure 4:**
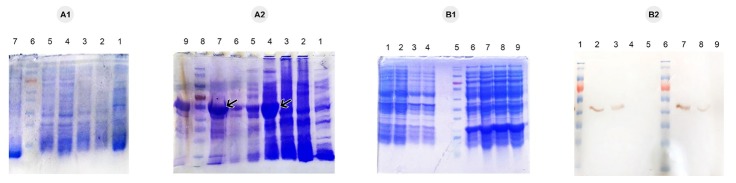
(**A1** and **A2**) SDS-PAGEs of GnRH-DFF40 protein expression before (Panel A1) and after (Panel A2) double-colony selections. Arrows indicate the expected protein band ~42 kDa. Panel A1 shows three different colonies before colony selection in HCDI (high cell density IPTG induction) (lanes 1-3) and AIM (autoinduction) (lanes 4, 5, 7) methods. Lane 6 shows molecular weight marker. Panel A2 shows expression results of three different colonies selected from the double-colony selection in HCDI method (Lanes 1–3) and other five selected colonies in AIM (lanes 4-7 and 9). Lane 8 indicates molecular weight marker. The colony gave a higher protein production was selected for optimization condition in autoinduction (AIM). (**B1** and **B2**) SDS PAGE and western blot analysis of recombinant GnRHDFF40 expression (with ~42kDa molecular weight) in AIM and HCDI methods. B1. Lane 1 & 2: non- induced samples as control in AIM medium at 37°C and 25°C respectively, lane 3 & 4: non-induced samples (without IPTG addition) as control in HCDI at 25°C and 37°C respectively, lane 5: molecular weight marker, lane 6 & 7: induced samples in HCDI at 25°C and 37°C respectively, lane 8 & 9: induced samples in AIM at 37°C and 25°C respectively. B2. Lane 1: molecular weight marker, lane 2 & 3: GnRH-DFF40 expression in AIM at 37°C and 25°C respectively, lane 4 & 5: non- induced samples as control in AIM medium at 25°C and 37°C respectively, lane 6: molecular weight marker, lane 7 & 8: GnRHDFF40 expression in HCDI method at 25°C and 37°C respectively, lane 9: non-induced samples (without IPTG addition) in HCDI at 37°C.

SDS-PAGE and western blot analysis using anti-His tag antibody revealed that chimeric protein was successfully expressed by these two methods under the conditions mentioned above. As demonstrated in Figure 3C and D, pH monitoring at 25°C and 37°C showed an optimum pH range about 6.5-7.5, 21 hours after incubation in both methods without any detrimental effects on cell growth. The pH variations at 25°C was less than 37°C in both methods at the same time points. Besides, it was evident from Figure 3C and D that the pH fluctuations were relatively low in AIM compared to HCDI method at both temperatures and at the same time period. It may be due to the existence of higher buffer capacity in AIM such as succinate. Although these alterations were closer together in both methods at 37°C. Figure 4B1 and B2 and Table 2 showed the effect of temperature on protein expression. As seen, expression of the recombinant protein at 25ºC is very high in both methods, although the amounts were somewhat higher in AIM method. These results demonstrated that protein production at a lower temperature increased the amount of soluble recombinant GnRH-DFF40 significantly. Gupta and co-workers revealed that at lower temperatures, the yield of recombinant protein is significantly higher due to enhanced protein solubility, decreased protein misfolding, improper formation of disulfide bonds and inclusion body formation.^26^ One another reason is that since bacterial growth at 37°C is more rapid and as a result cell growth also reaches higher density in a short time period, therefore the culture medium becomes acidic faster and the rate of plasmid loss increases. In general, AIM with 21 hours incubation time and at 25°C temperature is a more efficient method for producing a higher level of recombinant GnRH-DFF40 protein.

### Genetic stability of recombinant plasmid and double colony selection

One major drawback for no or low level of protein expression in growth media is plasmid loss that affected by various factors like the medium compositions, pH variations, and antibiotic degradation in medium.^27^ To resolve these problems, double colony selection to determine the high-level expression-colonies with high plasmid stability index was carried out. The plasmid stability in different methods was determined by calculating the plasmid stability index as shown in Figure 2B. These results demonstrated that only the recombinant plasmid containing synthetic GnRH-DFF40 in minimal AIM and HCDI were stably inherited with the plasmid stability index of 98% and 96%, respectively (Figure 4A1 and A2). These results clearly indicated that in each culture of other examined methods, a significant portion of the plasmid carrying cells is lost and an increase in cell density caused by an overgrowth of plasmid-free cells. Chen and colleagues indicated that pH decrease during the growth phase results in degradation of kanamycin and subsequently plasmid instability and significant decreasing of the protein amount in even a rich media.^28^ As suggested by Studier,^29^ we performed expression in several small volumes (5 × 50 mL) instead of a single large volume (250 mL) and a reproducible high level of soluble GnRH-DFF40 achieved. Moreover in all conditions indicated in Figure 2B, there is a higher tendency for plasmid stability containing synthetic fusion than that of native fusion form. Because synthetic form has a higher similarity with the genetic structure of *E. coli* and therefore higher compatibility for inheritance to bacterial progenies during the growth phase.^17^

### Determination of optimum conditions in AIM method

**Figure 5:**
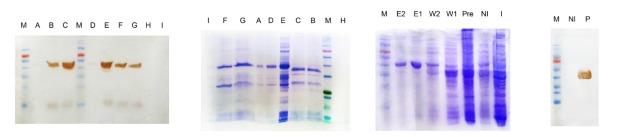
**(A)** SDS PAGE and western blotting analysis for determining of GnRH-DFF40 expression before purification in all optimization conditions of AIM method. Letters A to I indicate the A to I conditions demonstrated in Table 2B, M indicates molecular weight marker. (**B**) SDS PAGE and western blot analysis after purification of the best condition in the AIM method (condition E in Table 2B). M, E2, E1, W2, W1, Pre, NI and I in SDS PAGE analysis represent respectively molecular weight, elution 2, elution1, wash2, wash1, prewash, non-induced lysate, and induced lysate fractions obtained during the purification process of condition E in Table 3. In western blot analysis M, P and NI indicate molecular weight markers, purified GnRH-DFF40 and non-auto-induced sample respectively prepared based on condition E in Table 3. The purified proteins had a high purity and were visualized as single bands migrating at 42 kDa on SDS- polyacrylamide gel electrophoresis and western blotting.

An important step for high-level protein production using AIM method is the optimization of critical factors that mainly affect protein expression conditions such as glucose and lactose concentration. The results of different concentrations of glucose (0.05% to 0.2%) and lactose (0.2% to 0.7%) on cell density and protein yield using the highest expression colony obtained from double selection were demonstrated in Table 3. As mentioned by Faust et al, glucose and lactose sources are important in obtaining the high protein yields.^30^ After protein purification and determination of its concentration in all optimization conditions, the protein concentrations were high in B, C and E, intermediate in F and G and low level in A, D, H and I conditions. Each optimization condition was tested in three independent experiments with negligible differences in cell density and protein concentration. SDS page and western blot analysis of all optimization conditions have been illustrated in Figure 5A. Following determining the best optimized condition, a reproducible very high level of soluble GnRH-DFF40 yield (528.3 mg/L) was achieved using 0.1% w/v^-1^ of glucose and 0.5% w/v^-1^ of lactose concentrations (condition E in Table 3). AIM also has other advantages over HCDI method in terms of heterologous protein production including no need for OD monitoring and adding an inducer. Furthermore, it is a simple, effective and economical technique.^31^ On the other hand, induction with IPTG stresses the cell growth, as IPTG is mentioned to be noxious at a higher dose and during the long induction time but lactose can be metabolized in *E. coli*, into glucose and galactose.^26^ Due to these superiorities, AIM was used for the optimization of protein production. The BL21 derived strains unable to metabolize galactose. Hence, upon its accumulation in the cells, galactose can weakly bind to the lacI repressor protein at high concentrations, and induce a high level of protein production, therefore even after lactose utilization, induction can be continued by galactose.^32^ The inducing influence of remaining galactose could elucidate our observation that specific target protein production in BL21 (DE3) was maintained at a high level with lactose concentration of only 0.5 %wv^-1^.^25^

**Table 3:** Optimization of AIM method

**Condition**	**Glucose concentration** ** (%wv** ^-1^ **)**	**Lactose concentration** ** (%wv** ^-1^ **)**	**Cell density** ** (OD600)**	**Average amount of Protein concentration** **(mg/L)**
A	0.05	0.2	7.1±0.50	35.165‏±3.5
B	0.05	0.5	8.3±0.45	328.483±8.9
C	0.05	0.7	8.8±0.60	477.403±9.2
D	0.1	0.2	7.5±0.72	52.643±3.8
E	0.1	0.5	9.1±0.60	528.301±9.4
F	0.1	0.7	7.9±0.75	236.513±8.2
G	0.2	0.2	7.6±0.65	187.252±7.6
H	0.2	0.5	5.8±0.20	0
I	0.2	0.7	5.45±0.45	~0

In all optimization conditions of AIM method, a 100 mM phosphate and 0.5% glycerol was used for maintaining buffering capacity during glucose depletion. To enhance the buffering capacity against decreasing pH, organic acids with relatively high pK such as succinate was found to be effective. Also instead of acting simply as a buffer, succinate is metabolized near the glucose discharge during growth and results in bacterial medium reaches to higher cell mass and a higher pH than in the lack of succinate.^28,31,33^

### Recombinant protein purification

Under native conditions and using a Ni-NTA affinity column, the recombinant fusion protein His-tagged GnRH-DFF40 was successfully purified in a soluble form. Figure 5B demonstrated the SDS-PAGE and western blot analysis after protein purification only for the best optimized condition (condition E). Analysis of different fractions obtained during purification by SDS-PAGE showed an intense single band in elution fraction with molecular weight about 42 kDa corresponding to the GnRH-DFF40 fusion protein. Western blotting also verified the presence of this single band (~42 kDa) in elution after detection by an anti-His monoclonal antibody. This purification method prevents the cumbersome refolding processes of denaturing method that lead to extremely poor efficiency, particularly for proteins with several disulfide bonds.

## Conclusion

In the cases of “difficult-to-express” proteins or toxic protein production in *E. coli* such as GnRH-DFF40, the stability of recombinant plasmid-carrying cells, pH maintenance and medium composition is important. Therefore, the high-cell-density expression methods such as AIM and HCDI protocols may have a major superiority to the other conventional methods. The high plasmid stability, cell viability, convenience, efficiency, and economical cost have converted AIM to an ideal method for providing protein yields higher than the routine IPTG-induction protocols. Optimization of these methods in bacterial expression conditions could enhance the yield of proteins and shed lights for industrial applications in the future.

## Ethical Issues

Not applicable.

## Conflict of Interest

The authors declare that they have no conflict of interest.

## Acknowledgments

The present article was extracted from a Ph.D. thesis and financially supported by Shiraz University of Medical Sciences Grant No, 94-7580.
